# Evolutionary genetics of genotype H1 measles viruses in China from 1993 to 2012

**DOI:** 10.1099/vir.0.066746-0

**Published:** 2014-09

**Authors:** Songtao Xu, Yan Zhang, Pierre Rivailler, Huiling Wang, Yixin Ji, Zhu Zhen, Naiying Mao, Chongshan Li, William J. Bellini, Wenbo Xu, Paul A. Rota

**Affiliations:** 1 WHO Regional Reference Laboratory for Measles for the Western Pacific Region, National Institute for Viral Disease Control and Prevention, China Center for Disease Control and Prevention, Beijing 102206, PR China; 2 Division of Viral Diseases, Centers for Disease Control and Prevention, Atlanta, GA, USA; 3 Shanghai Center for Disease Control and Prevention, Shanghai City, PR China

## Abstract

Virologic surveillance is a critical component of measles management. One of the criteria for verification of elimination of endemic measles is genetic analysis of wild-type viruses to demonstrate lack of an indigenous genotype. Measles is yet to be eliminated in China, and genotype H1 has been detected continuously since virologic surveillance was initiated in 1993. Virologic surveillance has been very active in China, providing a unique opportunity to conduct a detailed study of the evolution of a single, endemic genotype over a timespan of nearly two decades. Phylogenetic analysis performed on the 450 nt coding sequence for the C-terminal 150 amino acids of the nucleoprotein (N-450), fusion (F) gene and haemagglutinin (H) gene confirmed the continued circulation of genotype H1 viruses for 19 years. No evidence of selective pressure for the H protein was found. The substitution rates ranged from 0.75×10^−3^ substitutions site^−1^ year^−1^ for H to 1.65×10^−3^ substitutions site^−1^ year^−1^ for N-450. The time of most recent common ancestor (TMRCA) for genotype H1 was estimated as approximately 1985 (95 % highest probability density, 1979–1989). Finally, the overall diversity of measles sequences from China decreased from 2005 to 2012, coincident with a substantial decrease in measles cases. The results suggest that detailed evolutionary analyses should facilitate the documentation of eventual measles elimination in China. Moreover, the molecular approaches used in this study can be applied in other countries approaching measles elimination.

## Introduction

Measles is a highly contagious disease characterized by high fever, cough and a maculopapular rash. The causative agent, measles virus (MeV), has a single-stranded, negative-sense RNA genome. MeV belongs to the genus *Morbillivirus* within the family *Paramyxoviridae*. The MeV genome is 15 894 nt in length, and contains six genes encoding nucleoprotein (N), phosphoprotein (P), matrix (M), fusion (F), haemagglutinin (H) and polymerase (L). The H and F proteins play key roles in viral entry and are the main targets of virus-neutralizing antibodies (Griffin & Bellini, 1996).

A safe and effective vaccine is available to control measles. Countries and regions with high vaccination coverage have successfully eliminated the transmission of endemic MeV. The World Health Organization (WHO) Region of Americas achieved measles elimination in 2002. The remaining WHO regions have established measles elimination goals for 2015–2020 (Anonymous, 2013; WHO, 2012a). Global vaccination programmes have achieved major reductions in measles-related mortality. Between 2000 and 2012, estimated global deaths due to measles decreased by 78 %, from 562 400 to 122 000, both historically low levels (Perry *et al.*, 2014). In 2005, China set a goal of measles elimination by 2012, and is currently working to achieve this aim by 2015 according to the regional plan of the Western Pacific Region.

Documentation of the interruption of MeV transmission is an essential criterion to confirm elimination, and laboratories play a key role in this process by providing virologic surveillance data (WHO, 2012a). The Chinese Measles Laboratory Network (CMLN), introduced in 2001 in China, is composed of one national, 31 provincial and 331 prefectural laboratories. The National Measles Laboratory performs genetic characterization of MeVs isolated in all the provinces.

Although MeV is monotypic, sequence differences between wild-type viruses have led to the designation of eight clades and 24 genotypes (Rota *et al.*, 2011; WHO, 2012b). Genotype H1 was identified as the endemic genotype in China with the initiation of viral surveillance in 1993 (Ji *et al.*, 2009, 2010; Xu *et al.*, 1998; Zhang *et al.*, 2007, 2008). While genotype H1 viruses have spread to other countries, this genotype is endemic only in China. The introduction of virologic surveillance in China has facilitated the genotyping of several thousand MeVs to date. Sequences from the genotype H1 viruses in China form two distinct clusters, previously designated cluster 1 and cluster 2 (Zhang *et al.*, 2007, 2008, 2009). Here, we took advantage of the unique opportunity to study the evolutionary genetics of a single, endemic and extensively characterized genotype in China over a nearly 20-year timespan. During this time, the incidence of measles in China declined steadily due to expanding vaccination programmes.

## Results

### Phylogeny of N, F, and H genes of genotype H1 MeVs from China

**Fig. 1.**
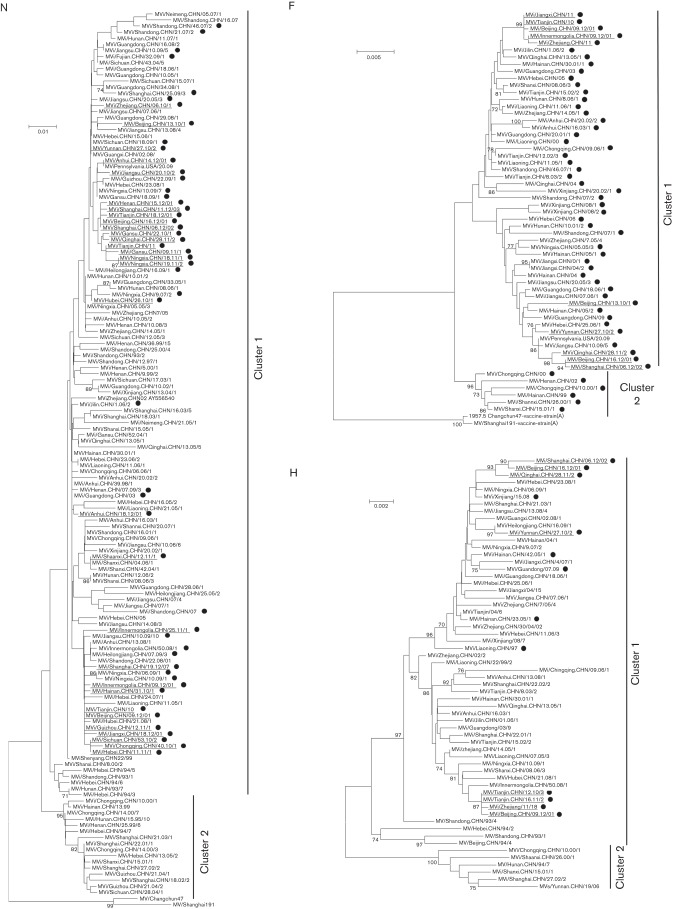
Phylogeny of N, F and H genes of genotype H1. Neighbour-joining phylogenetic trees were reconstructed using mega5. Sequences generated in this study are indicated with black circles. The names of viruses collected since 2010 are underlined. Groups corresponding to clusters 1 and 2 and bootstrap values greater than 70 % are indicated. Phylogenetic trees for N and F genes were rooted to genotype A Chinese vaccine strains Changchun47 and Shanghai191. GenBank ID numbers for the sequences used in the N, F and H datasets are presented in Table S1.

Among the 63 new viruses analysed, 33 had been collected since 2010 (Fig. 1). Phylogenetic analysis of the 450 nt sequence coding for the C-terminal 150 amino acids of N (N-450) showed that all new sequences are clustered within genotype H1, cluster 1. Phylogenetic analyses of the H and F genes revealed a similar tree topology, with cluster 1 containing the most recent sequences (Fig. 1). Interestingly, the H sequences of cluster 1 could be further divided into two subclusters, each supported by significant bootstrap values (>80 %). Viruses collected from the six different Chinese regions were classified into both subgroups, indicating no geographical restriction of subgroups (Fig. 1). Identification of positively selected sites within predicted protein sequences for H and F with fixed-effects likelihood (FEL) and internal FEL (IFEL) did not support the presence of positive selection for either (data not shown), consistent with previous observations (Rima *et al.*, 1997).

Two hundred and sixty-three sequences from 178 viruses of genotype H1 MeVs collected in all six regions of mainland China (Fig. S1, available in the online Supplementary Material) and 30 of 31 provinces were analysed. In addition, sequence data for all three genes were available from most provinces (Table S1). As expected, N-450 sequences were widely available, since N sequences are used for routine genotype identification. The oldest genotype H1 sequence was from a virus isolated in 1993 (MVi/Shandong.CHN/93/1-AF045207). Since then, viruses have been collected each year (Table S1). Although the dataset contained sequences collected over two decades, only a few sequences were available from 1993 to 1998. The number of available sequences increased in 2005 as a result of increased emphasis on virologic surveillance in China. To accurately estimate the time of most recent common ancestor (TMRCA) for genotype H1, the dataset needed to incorporate sequences of viruses collected before the initial detection of genotype H1. Therefore, sequences of the WHO reference strains collected between 1954 and 1993 were included in the final dataset (Table S2).

### Estimation of TMRCA for genotype H1 MeVs

Sequences from the N-450, H, and F genes were analysed. The H gene dataset appeared the most informative, since the entire 1854 nt coding region was included. The F sequences also included the entire coding region, but temporal distribution of the F dataset was limited compared to that of H. Accordingly, we performed the analysis on H sequences and used data from N and F genes to validate these results.

**Fig. 2.**
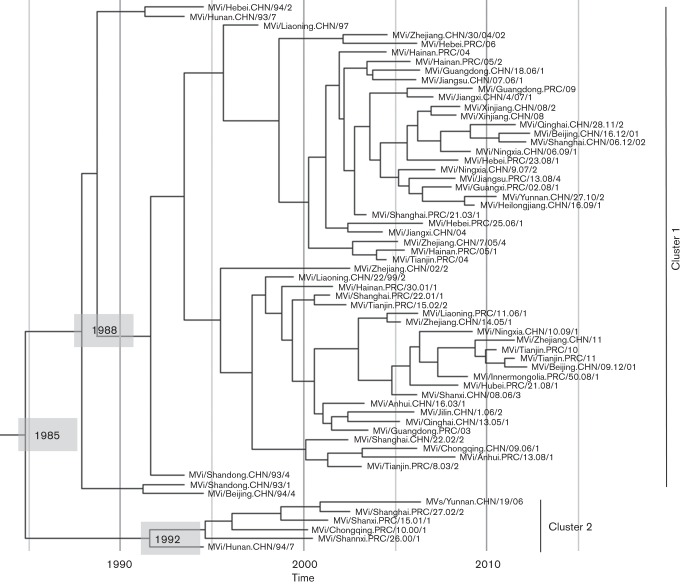
Maximum Clade Credibility (MCC) tree of H gene sequences of genotype H1 viruses from China. TMRCAs of interest are shown (see Table 1). Cluster 1 and cluster 2 are indicated. The complete MCC tree, featuring 95% HPDs node bars and posterior probabilities for the entire dataset is shown in Figure S3.

A Bayesian coalescent method was used for H sequence analysis to estimate TMRCA of the H1 genotype, as well as clusters 1 and 2 within the H1 genotype. The TMRCA of the H1 genotype (Table 1, Fig. 2) was estimated as approximately 1985 [95 % HPD (Highest Probability Density), 1979–1989]. The oldest genotype H1 virus analysed was collected in 1993. TMRCA suggests that genotype H1 viruses had been circulating in China at least 10 years before identification with virologic surveillance. Cluster 1 diverged at around 1988 (95 % HPD, 1984–1991) whereas cluster 2 appeared a few years later (95 % HPD, 1987–1994). Based on currently available data, cluster 2 appears to be inactive and cluster 1 viruses are widely distributed throughout China. Comparable date estimates were found with the N-450 and F datasets (Table 1).

**Table 1.** Nucleotide substitution rates and TMRCA for N-450, F and H genes of genotype H1 viruses from China

		MeV gene		
		H	F	N
Substitution rate*		0.75 (0.51–0.92)†	1.07 (0.54–1.75)	1.65 (1.19–2.11)
TMRCA	H1	1985 (1979–1989)	1989 (1978–1996)	1987 (1982–1994)
	Cluster 1	1988 (1984–1991)	1994 (1987–1998)	1989 (1985–1992)
	Cluster 2	1992 (1987–1994)	1994 (1987–1998)	1992 (1983–1994)

* Expressed as 10^−3^ substitutions site^−1^ year^−1^.

† Mean value is indicated first, followed by 95 % HPD in parentheses.

BEAST software was used to calculate nucleotide substitution rates for the N-450, F and H genes. The mean substitution rate for the H gene was estimated at 0.75×10^−3^ substitutions site^−1^ year^−1^ (95 % HPD, 0.51×10^−3^– 0.92×10^−3^). A similar range was estimated for the F gene with 95 % HPD of 0.54×10^−3^– 1.75×10^−3^ substitutions site^−1^ year^−1^. Notably, the substitution rate for N-450 was approximately twice as high with 95 % HPD of 1.19×10^−3^–2.11×10^−3^ substitutions site^−1^ year^−1^ (Table 1).

**Fig. 3.**
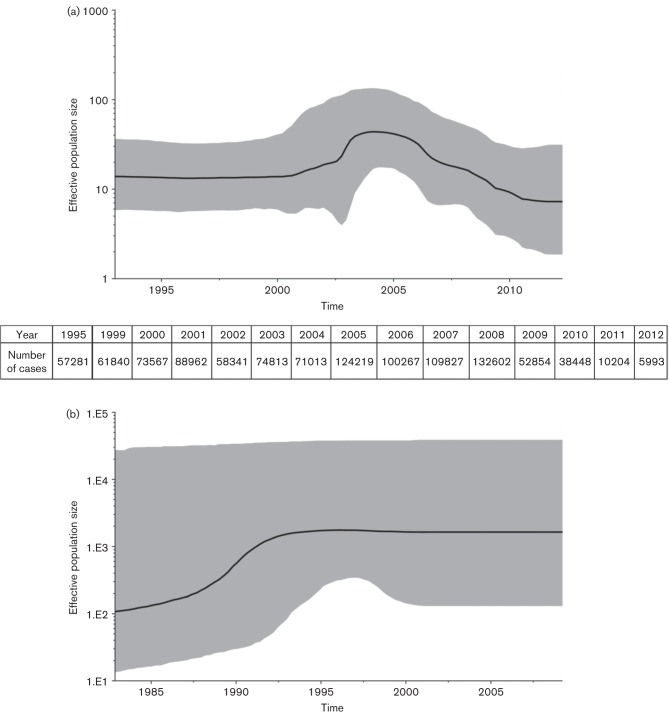
Bayesian skyline plots showing changes in the genetic diversity of H genes from genotypes H1 and B3 over time. Panel (a) shows sequence diversity for the H gene of genotype H1 viruses from China isolated between 1993 and 2012. The inset table shows the total number of measles cases reported in China over selected years. Panel (b) shows the diversity of H gene sequences for genotype B3 viruses detected in Africa. For both graphs, highlighted areas correspond to 95 % HPD intervals.

A Bayesian skyline plot was generated for the H gene dataset in order to analyse the genetic diversity of Chinese sequences over time (Fig. 3a). The graph has three distinct phases. From 1993 to 2000, minimal change in genetic diversity was evident, probably due to the limited number of viral sequences available. From 2000 to 2005, genetic diversity increased, consistent with the expansion of virologic surveillance in China during this time. Finally, from 2005 to 2012, genetic diversity steadily decreased, even though the number of sequences analysed was the same or greater than that for previous years (Figs S2a, 3a). The decrease in genetic diversity coincided with a significant reduction in the number of reported measles cases in China. A similar analysis was performed on H sequences of genotype B3 viruses of African origin (Fig. 3b, Table S3). The B3 skyline plot disclosed an increase in diversity from 1980 to 1992, possibly due to increased surveillance. From 1992 to 2012, the graph showed no change in diversity over time. Thus, it appears that the decrease in genetic diversity of genotype H1 reflects the decline in reported cases due to effective vaccination campaigns.

## Discussion

In this investigation, we had the unique opportunity to study the evolutionary genetics of a single, endemic and well characterized MeV genotype (H1) in China over a nearly 20-year timespan. Virologic surveillance began in China in 1993, and expanded significantly after 2005 to meet national programme goals. Currently, virologic surveillance is conducted by the National Measles Laboratory at the China Centers for Disease Control and Prevention, which oversees a network of more than 300 laboratories. From 2005 to 2012, the incidence of measles in China has decreased steadily due to expanding vaccination programmes.

Analysis of the H genes for genotype H1 viruses led to estimation of TMRCA for genotype H1 as around 1985, with divergence of two clusters (cluster 1 in 1987 and cluster 2 in 1991). Viral strains in cluster 2 have not been detected since 2005, and this lineage is presumably inactive, which is not an unusual occurrence for measles genotypes. Among the 24 measles genotypes recognized by WHO, only eight have been detected through global surveillance in the past five years (WHO, 2012b). Viruses in cluster 1 have been prevalent in mainland China since at least 1987. Our TMRCA estimates for the N and F genes were in close agreement with that for the H gene, even though the N and F datasets were limited by the short sequence length for N and incomplete representation for F.

The main focus of the current study was to analyse the evolution of a single, endemic genotype in a country with a strong surveillance system, with a view to limiting environmental effects and minimizing surveillance bias. Our analysis estimated appearance of the H1 genotype at around the mid-1980s, consistent with previous data on Asian genotypes D3, D5, D9 and H1 (Saitoh *et al.*, 2012) showing that H1 diverged in 1977 (95 % confidence interval, 1968 and 1984). Analysis of a limited number of Chinese N gene sequences led to TMCRA estimation for genotype H1 of around 1975 (Wei *et al.*, 2012). Previous studies also confirmed that the most reliable estimates of TMRCA are provided by analysis of the H gene, with N sequences giving comparable estimates (Pomeroy *et al.*, 2008; Saitoh *et al.*, 2012).

Studies on the molecular evolution of MeV are limited, since relatively few MeVs from the pre-vaccine era are available for genotyping. The majority of these older sequences are genotype A, which may have been distributed globally in the 1950s, 1960s and 1970s. The Chinese vaccine strain, S191, a member of genotype A, was isolated from a child in 1960 in Shanghai. Routine isolation and genotyping of wild-type MeVs did not begin on a global scale until the mid-to-late 1990s following the global measles resurgence in the late 1980s, and very few wild-type isolates are available from any part of the world before 1990.

RNA viruses, such as MeV, have the ability to undergo rapid genetic change (Domingo & Holland, 1997). These viruses evolve at an approximate rate of 10^−3^ nucleotide substitutions site^−1^ year^−1^ (Duffy *et al.*, 2008). For example, the capsid gene of GII.3 norovirus is reported to evolve at 4.2×10^−3^ substitutions site^−1^ year^−1^ (Mahar *et al.*, 2013), the HN gene of human parainfluenza virus type 1 at 0.77×10^−3^ substitutions site^−1^ year^−1^ (Mizuta *et al.*, 2011), and the G glycoprotein gene of human respiratory syncytial virus at 4.68×10^−3^ substitutions site^−1^ year^−1^ (Pretorius *et al.*, 2013). In this report, we calculated the nucleotide substitution rates for genotype H1 MeVs in China. The substitution rate calculated for the H gene of genotype H1 (0.75×10^−3^ substitutions site^−1^ year^−1^) was slightly higher than that of 0.65×10^−3^ substitutions site^−1^ year^−1^ reported by Pomeroy *et al.* (2008). A more recent study determined the substitution rate for the H gene of genotypes D3, D8, D9 and H1 as 0.56×10^−3^ substitutions site^−1^ year^−1^ (Saitoh *et al.*, 2012), which was similar to ~0.4×10^−3^ substitutions site^−1^ year^−1^ calculated for genotype D3 viruses circulating in Europe in the early 1990s and 0.34×10^−3^ substitutions site^−1^ year^−1^ for sequences from cases of subacute sclerosing panencephalitis (Rima *et al.*, 1997; Woelk *et al.*, 2002). Here, we estimated a rate of 1.65×10^−3^ substitutions site^−1^ year^−1^ for N-450, consistent with 1.12×10^−3^ substitutions site^−1^ year^−1^ calculated based on a dataset containing multiple genotypes, including H1 (Wei *et al.*, 2012). The substitution rate for N-450 was slightly higher than that of 0.86×10^−3^ substitutions site^−1^ year^−1^, which was calculated based on sequences for the entire coding region of the N gene (Pomeroy *et al.*, 2008).

Skyline plots showed that genetic diversity of genotype H1 MeVs in China decreased after 2005, while that of H genes of genotype B3 viruses from Africa remained constant. The decrease in genetic diversity in China coincided with a significant reduction in the number of reported measles cases. Thus, the decreased genetic diversity of genotype H1 possibly reflects the decline in measles due to effective vaccination campaigns. Of course, vaccination campaigns are also ongoing in Africa, leading to a dramatic drop in measles-associated mortality. However, vaccination coverage in most African countries is still considerably lower than that in China, and genotype B3 viruses continue to circulate widely as the endemic genotype in most sub-Saharan African countries (WHO, 2012b). Coalescent analyses were recently used to document the effectiveness of hepatitis B vaccine in the Netherlands (Hahné *et al.*, 2013) and antiviral therapy for hepatitis C virus in Egypt (Stadler *et al.*, 2013). The analytic approach described in the current report may provide a means for countries to use data from virologic surveillance for documenting progress towards measles elimination.

## Methods

### 

#### Virus isolation and sequencing.

Previously uncharacterized MeVs were sequenced to generate 52 N, 13 H, and 55 F sequences. B95a cells before June 2004 and after July 2004 (Kobune *et al.*, 1990) and Vero/hSLAM cells (Ono *et al.*, 2001) were used to isolate MeV. Viruses were passaged no more than twice. Both cell lines express a receptor for wild-type measles virus, and therefore passage in these cells would not require amino acid substitution associated with adaptation. RNA extraction, amplification and sequencing of N and H genes were performed as described previously (Pond & Frost, 2005; Zhang *et al.*, 2007). The complete coding region (1662 nt) of the F gene was amplified with the sequence-specific primers, MeV-F-for: GAA TCC CAG AAT CAA GAC TCA TC and MeV-F-rev: CAT TTG TGT TTC AAG AGT TG, using the SuperScript^TM^III One-Step reverse transcription (RT)-PCR kit (Invitrogen). The sequencing primers used were MeV-F-seq1: CCT CTG CTC CAA GAA TGC CTC CGG, MeV-F-seq2: TCT GTA TAG TAT CTG AGC AAT TTG AG, and MeV-F-seq3: TGC ACA ATT GGC TAT TAG G. Metadata, including GenBank ID numbers, are available in Table S1.

#### Sequence datasets used for analysis.

Three sequence datasets, one for each gene, were established. The N and H gene datasets were derived mostly from previously reported sequences and complemented with those of viruses collected after 2008 (for N) and 2009 (for H). In order to avoid oversampling due to large outbreaks, similar sequences (less than 1 % nucleotide divergence) from viruses collected at the same times and locations were deleted. To obtain a meaningful estimation of the TMRCA for genotype H1, sequences of viruses collected before the estimated appearance of genotype H1 (1993) were added to the dataset. The F gene sequence dataset was more straightforward, as all available F gene sequences were included. Metadata for all three datasets are shown in Tables S1 and S2. The final datasets contained 170 N (N-450) sequences (144 genotype H1 and 26 from non-H1 WHO reference strains), 86 H sequences (60 genotype H1 and 26 from non-H1 WHO reference strains), and 69 F sequences (57 genotype H1 and 12 from non-H1 WHO reference strains).

#### Sequence analysis and phylogeny inference.

Sequence alignments were generated with MUSCLE (Edgar, 2004) and edited with BioEdit (http://www.mbio.ncsu.edu/BioEdit/bioedit.html). Phylogenies were inferred with mega software (version 5) using the neighbour-joining method and a maximum composite likelihood model (Tamura *et al.*, 2011; Saitou & Nei, 1987). The reliability of phylogenetic inference at each branch node was estimated by the bootstrap method with 1000 replications using mega (Efron & Tibshirani, 1994). TMRCA was estimated using a coalescent-based Bayesian method implemented in BEAST (version 1.7.2) (Drummond *et al.*, 2012). The nucleotide substitution model was tested in jModelTest0.1 using Akaike information criterion (AIC) (Guindon & Gascuel, 2003; Posada, 2008). The best-fit model of nucleotide substitution for the three datasets was determined as GTR+I+G [general time reversible model (GTR); proportion of invariable sites (I); gamma distribution shape parameter (G)]. Initially, three molecular clock models were tested on each dataset: strict, uncorrelated exponential relaxed and uncorrelated lognormal relaxed clock. The Bayes factor test determined that the uncorrelated exponential relaxed clock (UCER) with eight rate categories was the best fit for each dataset (Drummond *et al.*, 2012). Therefore, only results using this model are shown. Three independent analyses were run for each dataset and combined using LogCombiner (Drummond *et al.*, 2012). Sixty to ninety million generations were run, and log and tree files saved to generate 10 000 trees, ensuring sufficient sample size (*n*>200) for each parameter of interest. Maximum clade credibility (MCC)-dated phylogenetic trees were summarized after the removal of 10 % sample burn-in with TreeAnnotator, and edited in FigTree version 1.3.1 (Drummond *et al.*, 2012; Rambaut & Drummond, 2010). Bayesian skyline plots depicting relative viral genetic diversity through time were generated for the H gene in TRACER (Drummond *et al.*, 2005). As a control, 63 H sequences of genotype B3 genotype were analysed via skyline plot (Table S3).

#### Selective pressure analysis.

To evaluate selective pressure on the H and F coding regions among the genotype H1 MeV strains, the rates of synonymous (dS) and non-synonymous (dN) substitutions were estimated with both FEL and IFEL methods using maximum-likelihood approaches available on the Datamonkey webserver (http://www.datamonkey.org/) (Pond & Frost, 2005). Positive (dN>dS) selections were predicted at a significance level (*P*-value) of <0.05.
